# Chaihu-Guizhi-Ganjiang Decoction is more efficacious in treating irritable bowel syndrome than Dicetel according to metabolomics analysis

**DOI:** 10.1186/s13020-022-00695-4

**Published:** 2022-12-14

**Authors:** Mingming Li, Jiawei Zhu, Xuan Liu, Zhiying Dong, Jigui Tang, Cian Zhang, Jianpeng Jiao, Jiani Chen, Fenghao Yin, Shi Qiu, Feng Zhang, Shouhong Gao, Zhipeng Wang, Xia Tao, Xiaoqiang Yue, Lianna Sun, Wansheng Chen

**Affiliations:** 1 School of Pharmacy, Shanghai University of Traditional Chinese Medicine, Shanghai, 201203 People’s Republic of China; 2 Department of Pharmacy, Second Affiliated Hospital of Naval Medical University, Shanghai, 200003 China; 3 Department of Traditional Chinese Medicine, Second Affiliated Hospital of Naval Medical University, Shanghai, 200003 China; 4 Department of Pharmacy, 905 Hospital of People’s Liberation Army Navy, Shanghai, 200050 China; 5 Traditional Chinese Medicine Resource and Technology Center, Shanghai University of Traditional Chinese Medicine, Shanghai, 201203 China; 6 Shanghai Key Laboratory for Pharmaceutical Metabolite Research, Shanghai, 200433 China

**Keywords:** Irritable bowel syndrome, Metabolomics analysis, Chaihu-Guizhi-Ganjiang Decoction, Dicetel, Carnitine

## Abstract

**Background:**

Chaihu-Guizhi-Ganjiang Decoction (CGGD) is a traditional Chinese medicine (TCM) prescription used to treat viral influenza. There is evidence that CGGD can be used to treat irritable bowel syndrome (IBS) but the potential mechanism of action and metabolites produced upon CGGD treatment remains elusive.

**Methods:**

Patients with IBS were treated with pinaverium bromide (Dicetel™) and then CGGD after a washout period of 1 week. Both treatments lasted for 30 days. The efficacy and changes of metabolites in plasma after the two treatments were compared. Plasma samples were acquired before and after each treatment, and untargeted metabolics analysis was performed.

**Results:**

Efficacy was measured according to the Rome IV criteria and TCM theory. Our results indicated that CGGD showed significantly better efficacy than Dicetel in the treatment of IBS utilizing each criterion. CGGD exerted greater effects on plasma metabolism than Dicetel. Dicetel treatment led to increased tryptophan metabolism (increased levels of 5-Hydroxyindoleacetaldehyde) and increased protein metabolism (increased levels of L-arginine). CGGD treatment significantly (p < 0.05) increased carnitine metabolism, with elevated levels of L-carnitine and acylcarnitine in plasma. Such changes in these metabolites could exert effects against IBS by improving gastrointestinal motility and suppressing pain, depression, and inflammation.

**Conclusions:**

CGGD appeared to be more efficacious than Dicetel for treating patients with IBS. The findings provide a sound support for the underlying biomolecular mechanism of CGGD in the prevention and treatment of IBS.

**Supplementary Information:**

The online version contains supplementary material available at 10.1186/s13020-022-00695-4.

## Background

Irritable bowel syndrome (IBS) is a debilitating, chronic, and highly prevalent disorder of gut–brain interaction [1, 2]. In routine clinical practice, IBS symptoms include recurrent disordered defecation and abdominal pain [3].

According to the Rome IV criteria, IBS is defined as that patients with IBS recurrent abdominal pain symptoms at least once a week on average [4]. The Rome IV criteria were derived by consensus from a multinational group of experts in disorders of gut–brain interaction [4].

IBS is a common disease with a prevalence of approximately 4.4–4.8% in the USA, UK, and Canada. IBS is more common in females and individuals younger than 50 years [5]. IBS is a highly burdensome condition for healthcare systems, families, and society worldwide. Direct medical costs attributed to IBS in the USA (excluding prescription and over-the-counter medications) have been estimated to be $1.5–$10 billion per year [6]. The direct and indirect costs related to IBS has been estimated to be up to ¥123 billion in China [7]. According to a meta-analysis of 80 clinical studies involving 260,960 individuals in 2012 [8], the IBS prevalence in China was 6.5%. The overall prevalence of IBS in the Chinese population was ≤ 10% in 2016, and the peak age of onset was 30–59 years. Female gender, alcohol drinking, history of intestinal infection, anxiety, depression, or food allergy, and certain living conditions are risk factors for Chinese IBS patients [8–11]. Therefore, the prevention and treatment of IBS is quite important.

The factors leading to susceptibility to IBS are diverse. Despite clearly established symptoms, the pathology of IBS is complicated and loosely understood. In general, it is believed that IBS is a result of a combination of multiple factors, including mental/psychological disorders, visceral hypersensitivity, intestinal infection, and disorders in gastrointestinal (GI) motility [12–14]. In routine practice, drugs such as pinaverium bromide (Dicetel™) and Alosetron™ play roles in intestinal movement. These medication diminish visceral hypersensitivity, improve emotions, and regulate intestinal flora. Dicetel and Alosetron are used commonly to relieve IBS symptoms [15–18]. Dicetel reduces the plateau phase of slow waves in the electrical activity of the smooth muscle cells, thereby inhibiting influx of calcium ions (Ca2 +) and preventing consequent contractions of intestinal smooth muscle [19, 20]. Therefore, Dicetel is recommended as a first-line therapy for short-term relief from IBS symptoms [21]. However, due to side effects over a long-term use of Dicetel and Alosetron, efficacious therapy of IBS warrants further investigation.

Traditional Chinese medicine (TCM) formulations have shown favorable effects in IBS treatment [22–24]. According to TCM theory, the etiology of IBS is attributed mainly to the weakness of Pi (spleen) and Wei (stomach) or damage to the Gan (liver) [7, 25], which is summarized as “Dan Re Pi Han” in TCM theory.

Recently, Chaihu Guizhi Ganjiang Decoction (CGGD) has been developed and used to treat liver/spleen deficiency, abdominal pain, and diarrhea [26]. CGGD is composed of Chinese thorowax, Ramulus cinnamomi, Rhizoma Zingiberis, root of Trichosanthes species, Scutellaria baicalensis, oyster shell, and Radix Glycyrrhizae Preparata. CGGD has been shown to have a promising effect against IBS, but its underlying pharmacological mechanism of action remains elusive.

Metabolomics is a comprehensive study of low-molecular-weight metabolites in biological systems, which offers extensive phenotypic information not available by genetic testing [27]. TCM theory focus on the diagnosis and treatment of diseases based on understanding of the overall dynamic state of patients. Hence, metabolomics analysis could provide more direct and important information for deciphering the pharmacological mechanism of action of TCM formulations [28]. Up to now, several studies of CGGD have been reported; however, the effect of CGGD on the plasma metabolome in IBS patients remains unclear.

In this study, we determined the effect of CGGD against IBS compared with that of Dicetel. Moreover, we analyzed the effect of CGGD on the plasma metabolome in IBS patients and explored the potential pharmacological mechanism of action of CGGD against IBS.

## Materials and methods

### Ethical approval of the study protocol

The study protocol was granted by the Biomedical Research Ethics Committee of Shanghai Changzheng Hospital (Shanghai, China) (No. 2019SL033). Written informed consent was obtained from all participants in the study [10].

### Study design

**Fig. 1:**
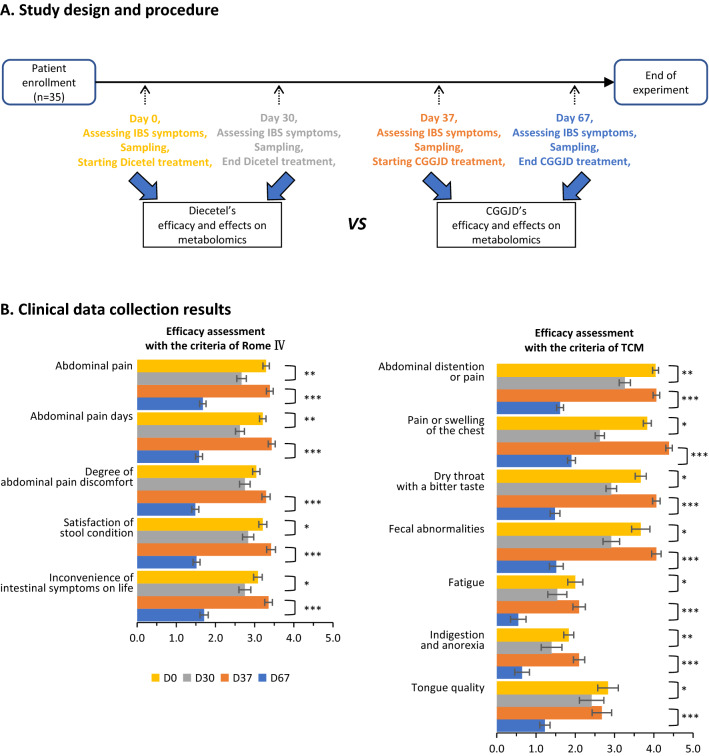
Experimental design and collection of clinical data. **A** Study design. Patients were enrolled for eligibility assessment based on inclusion criteria. Thirty-five patients were enrolled. “D0–D30” denotes from Day 0 (D0) to Day 30 (D30), and represents differences in patients before and after Dicetel treatment in terms of the Symptom Index score as well as collected blood samples before and after treatment. Eventually, 24 patients provided Discomfort Index scores before and after treatment. D37–D67 represents differences in patients before and after CGGD treatment in terms of the Symptom Index score and collected blood samples before and after treatment. In the middle of these two periods is a 1-week washout period. Thirty-one patients provided scores for the Discomfort Index before and after treatment. “D30 vs. D0” denotes the procedure of exploring the therapeutic effect of Dicetel and the effect of the human metabolome. “D67 vs. D37” refers to the procedure of exploring the therapeutic effect of CGGD and the effect of the human metabolome. **B** Clinical data. The numerical mean of the Symptom Index score and error bar, and P-value are shown. *Represents differences between different groups of the same target. *p < 0.05, **p < 0.01, ***p < 0.001. P-value was calculated by the Student’s t-test. Y-axis represents the symptom under different criteria. The X-axis represents the average Symptom Index score when taking different drugs at different stages. Criteria are divided into Rome IV and TCM. The Symptom Index score of patients under different criteria (Rome IV or TCM) is selected, followed by selected by different reference indicators under these two criteria (Additional file 1: Table S1). First, the enrollment time was recorded as “D0” and the Symptom Index was recorded. Second, after 4 weeks of treatment with Dicetel, the Symptom Index score of patients condition at this time was recorded as “D30”. Third, after the washout period (1 week), the Symptom Index score was recorded as “D37”. Finally, after 4 weeks of treatment with CGGD, the Symptom Index score was recorded as “D67”

This was a prospective study (Fig. 1A). Patients were treated with two drugs successively and divided into two groups: the positive drug group (Dicetel) and the CGGD control group. The duration of these two treatments was 1 month. A washout period of 1 week was setup to determine the Symptom Index score. The plasma samples of patients were collected across four time periods.

### Diagnosis of IBS

The diagnosis of IBS was based on the Rome IV criteria. The typical clinical manifestations of IBS are recurrent abdominal pain (≥ 1 day a week in the last 3 months) accompanied by changes in defecation frequency and fecal traits (appearance). Symptoms appear for ≥ 6 months and persist for the previous 3 months [29].

The diagnosis of IBS based on TCM theory was also used for efficacy evaluation. It was formulated according to the *Clinical research of new Chinese medicine and the consensus of TCM Diagnosis* [30] and *Treatment of irritable bowel syndrome* (2017) [31]. The main criteria were: (1) pain or swelling of the chest; (2) distension/pain in the abdomen, emotional relapse/aggravation; (3) fecal abnormalities; (4) dry throat with a bitter taste, or dizziness. Secondary criteria were: (i) indigestion and anorexia; (ii) fatigue; (iii) tongue quality. Patients with (3) and at least one of (1), (2), or (4) of the main criteria were diagnosed as having IBS. Patients with two features of secondary criteria and more than one of (1), (2) and (4) of the main criteria were assessed to have IBS.

### Inclusion criteria

The inclusion criteria were: (1) meeting the diagnostic criteria for IBS; (2) aged between 18 and 75 years old; (3) providing written informed consent to participate in the study.

### Exclusion criteria

The exclusion criteria were: (1) constipation in IBS; (2) patients with other chronic or serious diseases (e.g., heart disease, renal failure); (3) patients with other diseases that affect normal physician–patient communication; (4) combination of two or more diseases meeting the inclusion criteria; (5) long-term use of similar drugs used in our study.

### Elimination criteria

The criteria for elimination from this study were: (1) patients failing to receive medication as planned; (2) patients taking drugs other than the study drugs (including TCM formulations or drugs with anti-tumor effects); (3) treatment ceased due to suspected safety issues.

### Collection of plasma samples and treatment

Thirty-three patients aged 23–72 years diagnosed with IBS were enrolled from 2020 to 2021. The study lasted for 17 weeks as follows: Dicetel treatment (4 weeks), washout period (1 week), CGGD treatment (4 weeks), and follow-up (8 weeks). During the entire observation period, living conditions and dietary habits remain unchanged according to self-reporting by patients (Table 1).

**Table 1:** Basic information of patients

Age (years)	
< 50	30
50–59	4
60–69	2
> = 70	2
Sex	
Male	23
Female	15
Total	38

Patients were treated with Dicetel (50 mg, t.d.s., p.o. [with food]; Abbott Healthcare SAS, Paris, France). The treatment spanned 4 weeks, followed by 1 week of rest without treatment. Then, they were treated with CGGD (1 bag, t.d.s., p.o. [post-prandial]; Guangdong Yifang Pharmaceuticals, Foshan, Guangdong) for 4 weeks. Before and after each treatment, the Discomfort Index of patients according to TCM theory and the Rome IV criteria was obtained. The Discomfort Index was completed by patients and follow-up was conducted in the outpatient setting, inpatient setting, and by telephone consultation. Data input and verification were completed and checked by two individual researchers. Finally, clinical data were obtained for statistical analyses and clinical observations were recorded.

Simultaneously, plasma samples (5 mL) from each patient at D0 (before Dicetel treatment), D30 (after Dicetel treatment), D37 (before CGGD treatment), and D67 (after CGGD treatment) were collected. Samples were stored at − 80 °C for subsequent analyses.

### Metabolomics analysis

Untargeted metabolomic analysis was modified according to our previous work [32]. Sample pretreatment was carried out by protein precipitation. Ultra-high-performance liquid chromatography-quadruple-time of flight-mass spectrometry (UHPLC-Q-TOF–MS) was undertaken on a 1290 Series UHPLC system (Agilent Technologies, Santa Clara, CA, USA) coupled with a 6530 Accurate-Mass Q-TOF LC/MS system (Agilent Technologies) in positive electrospray-ionization mode (Dual Jet Stream; Agilent Technologies).

For chromatography analysis, metabolites separation was performed using a HSS T3 (3.5 μm, 2.1 × 100 mm) column (Waters, Milford, MA, USA), and the flow rate was maintained at 0.4 mL/min. The mobile phase A was water with formic acid (0.1% v/v), and mobile phase B was acetonitrile with formic acid (0.1% v/v). The temperature was maintained at 30 °C. The gradient started with 5% B, increased to 10% at 3.5 min, 40% at 6 min, 60% at 16 min, 80% at 20 min, 100% at 20.3 min, and a post-run of 5 min. The injection volume was 3 μL. The results of chromatography analysis are shown in the Additional files 2. 

For mass spectrometry analysis, the capillary voltage was 3500 V, and the nozzle voltage was 500 V. The gas temperature was set at 300 °C with a gas flow of 11 L/min and nebulizer pressure of 35 psi; and a sheath gas temperature was set at 300 °C with a sheath gas flow of 11 L/min. For MS acquisition, centroid data were acquired from 100 to 1100 m/z at 0.5-s intervals. For MS/MS acquisition, data were obtained from 0.33-s intervals with collision energy 0, 10, 20 and 40 eV. A reference solution (m/z 121.0509 and m/z 922.0098) was used to correct small mass drifts during the acquisition [33]. Quality control (QC) samples were injected at the beginning of the run and after every eight samples during sequence analysis to assess the analytical performance [34]. The basic information of results of mass spectrometry analysis are shown in the Additional files 3. 

### Statistical analyses

Semi-quantitative levels of the plasma metabolome were extracted by MS-DIAL 4.48 (http://prime.psc.riken.jp). All ions were normalized to the internal standard. Ion peaks with a coefficient of variation < 30% in QC samples and co-expressed with additional ions were filtered. The filtered ions were identified by MSFINDER 3.50 (http://prime.psc.riken.jp). Metabolites with the highest identification score encoded by the Human Metabolome Database (https://hmdb.ca) and METLIN (http://metlin.scripps.edu/) were selected for subsequent analyses. Then, data were processed through multiple steps: (1) quantile normalization, (2) log_2_ transformation, (3) Pareto Norm sequentially, (4) IQR filtering of metabolites with large within-group variances, and (5) data imputation using K-nearest neighbor [35]. Differential analyses were performed using limma [36]. All clinical covariates were adjusted. Gephi v0.9.2 (https://gephi.org) was used to generate a network based on the results of correlation analyses [37]. The Student’s *t*-test was used to screen CGGD- and Dicetel-related endogenous metabolites. Statistical analyses were carried out using R software (v3.6.3), and P < 0.05 was considered to indicate statistical significance. The code of R are shown in the Additional files 4, 5, and 6. 

## Results

### Beneficial effect of CGGD against IBS compared with Dicetel

CGGD showed significantly better efficacy than Dicetel against IBS, according to whether the Rome IV criteria or TCM theory. Efficacy assessment according to the Rome IV criteria is based on five criteria (Fig. 1B): Dicetel improved four of the symptoms and CGGD improved all five ones. In addition, for each criterion, a greater statistical significance in the above changes from CGGD was observed compared with Dicetel. The maximal, median, and minimal degree in improvement of all Rome IV criteria for Dicetel was 0.63, 0.38, and 0.29, and for CGGD was 1.90, 1.81, and 1.64, respectively.

For Dicetel, the least-improved criterion was “Inconvenience of intestinal symptoms on life” (average Symptom Index score decreased from 3.08 to 2.75). The most-improved criterion was “Abdominal pain” (average Symptom Index score decreased from 3.29 to 2.65).

For CGGD, the least-improved criterion was “Satisfaction of stool condition” (average Symptom Index score decreased from 3.42 to 1.52). The most-improved criterion was “Abdominal pain” (average symptom index decreased from 3.29 to 2.65).

Similar results were obtained using TCM theory. Efficacy assessment using TCM theory contains seven criteria (Fig. 1B): Dicetel and CGGD improved all of them. For each criterion, CGGD caused larger changes with greater statistical significance than Dicetel. The maximal, median, and minimal degree of improvement of all TCM-theory criteria for Dicetel was 1.21, 0.75, and 0.42, and for CGGD was 2.58, 2.45, and 1.45, respectively.

For Dicetel, the least-improved criterion was “Tongue quality” (average Symptom Index score decreased from 2.83 to 2.42). The most-improved criterion was “Pain or swelling of the chest” (average Symptom Index decreased from 3.83 to 2.63).

For CGGD, the least-improved criterion was “Indigestion and anorexia” (average Symptom Index score decreased from 2.10 to 0.65). The most-improved criterion was “Dry throat with a bitter taste” (average symptom index decreased from 4.06 to 1.48).

### Influence of Dicetel and CGGD on the plasma metabolome

A total of 643 plasma metabolites were identified using untargeted metabolome analysis. Among them, four categories of endogenous metabolites were related to the effect of Dicetel (significant difference between D30 vs. D0). The largest category was “organic acids and derivatives”, which contained metabolites such as N-acetyltryptophan, and L-arginine. The second largest category was “lipids and lipid-like molecules”, including metabolites such as malonylcarnitine (AC_4_-OH) and pimelylcarnitine (AC_6_-COOH). The third largest category was “organoheterocyclic compounds”, containing metabolites such as 5-Hydroxyindoleacetaldehyde (5-HIAA). In line with this, the plasma levels of most of these metabolites were also increased upon Dicetel treatment.

Seven categories of endogenous metabolites were related to the effect of CGGD (significant difference between D67 vs. D37). The largest category was “lipids and lipid-like molecules”, involving metabolites such as hydroxyisovaleroyl carnitine (AC_4_–CH_3_), 3-hydroxyoctanoyl carnitine (AC_8_–OH), phosphatidylethanolamine (35:0), 3-hydroxydecanoyl carnitine (AC_10_–OH), and phosphatidylcholine (40:7). The second largest category was “organic acids and derivatives”, including metabolites such as L-arginine. The third largest category was “organoheterocyclic metabolites”, which contained metabolites such as 5-HIAA. Consistently, the plasma levels of most of these metabolites were increased upon CGGD treatment.

According to principal components analysis (PCA) and partial least squares-discriminant analysis (PLS-DA) for these treatment effect-related metabolites, it was revealed that the CGGD-group samples showed better separation compared with the Dicetel-group samples (Fig. 1B). The first principal component (PC1) for the CGGD group and Dicetel group was 33.5%, 37.8% The PC1 of PLS-DA for the CGGD group and the Dicetel group was 32.6%, 36.5%, respectively.

### Analysis of enrichment of biological pathways on CGGD and Dicetel-related differential metabolites

**Fig. 2:**
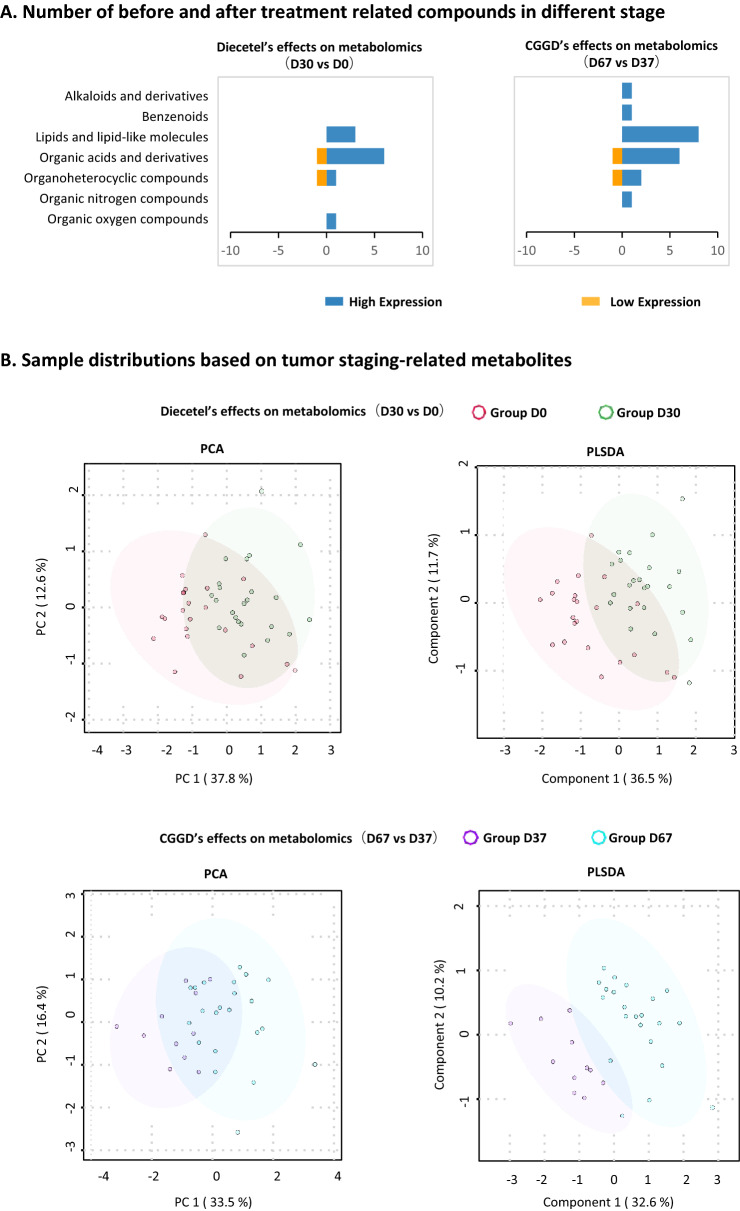
Differences in metabolites after treatment with Dicetel and CGGD. **A** Number of metabolites associated with a significantly altered treatment effect. Patients was divided into a Dicetel group (D30 vs. D0) and CGGD group (D67 vs. D37). The part of the axis that is > 0 indicates the number of metabolites whose concentration exhibits an increasing trend. The part of the axis that is < 0 indicates the number of metabolites whose concentration exhibits a decreasing trend. The ordinate expresses a simplified “super-class” of metabolites. The abscissa expresses the number of these simplified super-classes exhibiting high/low expression at different stages. These metabolites were divided into seven species according to their superclass from an Internet website (https://hmdb.ca/): “benzenoids”; “lipids and lipid-like molecules”; “organic acids and their derivatives”; “organoheterocyclic metabolites”; “organic nitrogen metabolites”; “organic oxygen metabolites”; “alkaloids and their derivatives”. **B** Sample distributions based on differences in efficacy-related metabolites. The positions of the different-colored spheres represent the distribution of different metabolites at different stages. PCA and PLS-DA plots using differential metabolites associated with treatment efficacy show different sample distributions in the Dicetel group and CGGD group

Differential metabolites were subjected to further analysis to reveal their biological function in a phenotypic context. In this way, we clarified the reason of the difference in efficacy between CGGD and Dicetel for treating IBS (Fig. 2).

**Fig. 3:**
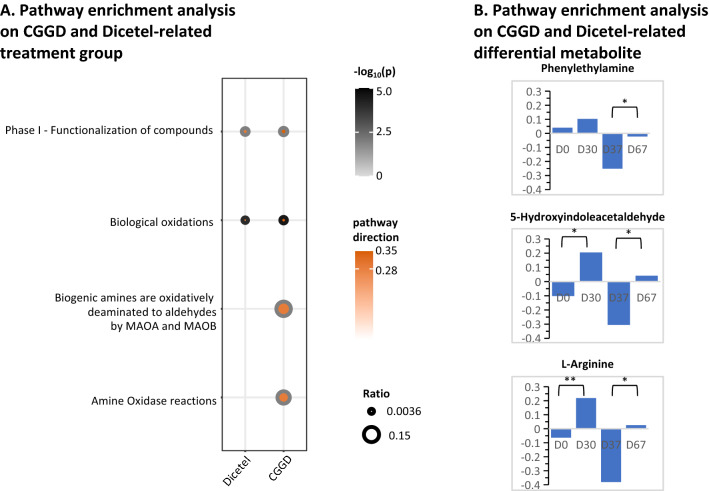
Analysis of biological pathway enrichment. **A** Biological pathway enrichment analyses in the CGGD group and Dicetel group. The p-value was set at 0.05. The dot border color represents the significance value of pathway enrichment. “Pathway weight” is defined as the number of entities found/total number of entities. The size of the dot represents the pathway weight. “Pathway direction” is the median log_2_ fold change (FC) of metabolites in the pathway (red = upregulated). *p < 0.05 for D67 vs. D37 and D30 vs. D0. **B** Biological pathway enrichment analyses in the CGGD group and Dicetel group according to differential metabolites. The evaluation criteria were identical to those shown in Fig. 1B. The number on the Y-axis and length of the column represent the concentration of the metabolite at this stage. The X-axis represents the different stages (D0, D30, D37, and D67)

Biological pathway enrichment was carried out using the Reactome Pathway Database (https://reactome.org/) for metabolites associated with efficacy in the Dicetel group and CGGD group (Fig. 3). Overall, the above metabolites enriched in pathways mainly regulate “Metabolism of amino acids and derivatives”. All enriched biological pathways were upregulated in the CGGD group and Dicetel group (Fig. 3A). It is noteworthy that “Phase I—Functionalization of metabolites” and “Biological oxidations” were the shared biological pathways between the CGGD group and Dicetel group.

Furthermore, the metabolites from the shared biological pathways between the Dicetel group and CGGD group were subjected to subsequent analysis. 5-HIAA and arginine were enriched in the CGGD group and Dicetel group. Phenylethylamine (PEA) was enriched only in the CGGD group.

### Correlation analyses between CGGD- and Dicetel-related differential metabolites

**Fig. 4:**
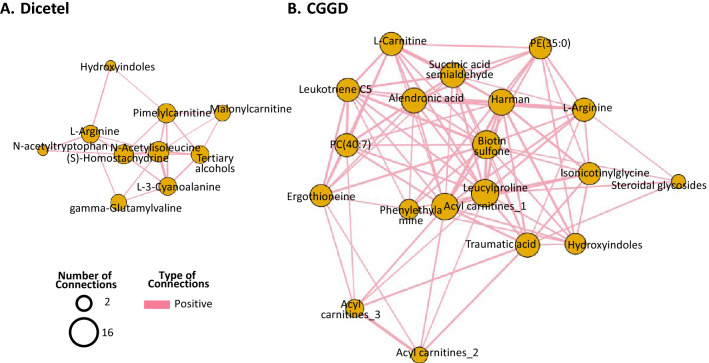
Correlation analyses. The correlation network reflects the correlation of compounds in the certain pathway. The metabolite–metabolite correlation network comprised only paired metabolites with a significant correlations (p ≤ 0.05). The degree of thickness, degree of edge, and diameter of the node show the importance of metabolites

Correlation analysis and a metabolite-metabolite network were performed to assess the associations between CGGD- and Dicetel-related differential metabolites. Correlation analysis demonstrated that the most correlated metabolites in the Dicetel group were N-acetyltryptophan, AC_6_-COOH, and arginine (Fig. 4A). The most correlated metabolites in the CGGD group were biotin sulfone, leucylproline, and AC_4_-CH_3_ (Fig. 4B). These highly correlated metabolites may have important roles in the pharmacological effect of Dicetel and CGGD against IBS.

## Discussion

### Conventional mechanism of action of IBS

Traditionally, the diagnosis of IBS has been based on identification of symptoms that correlate with several different syndromes associated with disorders such as IBS diarrhea, IBS constipation, functional diarrhea, functional constipation, chronic functional abdominal pain, or bloating [38]. Several peripheral and central mechanisms initiate disorders of GI motor and sensory functions leading to IBS symptoms [39]. The predominant pathophysiological mechanisms in IBS are abnormalities of gut smooth muscle, visceral hypersensitivity, and central nervous system (CNS) hypervigilance. IBS symptoms are not specific to a single etiologic mechanism but are manifestations of several peripheral mechanisms that perturb motor and sensory functions [39]. Our study lacked a healthy control group to elucidate directly the metabolomic basis of IBS, which should be conduct in the future. Nevertheless, anti-IBS drugs induced metabolomics changes in regulation of biological pathways based on abnormalities of gut smooth muscle and CNS hypervigilance. These biological pathways included the metabolism of tryptophan, arginine, and L-carnitine-regulated lipid metabolism (Additional files 2, 3, 4, 5, 6).

### Pharmacological mechanism of action of Dicetel in the body

Dicetel is a GI-selective antagonist of Ca^2+^ channels. It has highly selective spasmolytic activity in the GI tract. Dicetel helps to lessen the discomfort and abdominal pain associated with functional intestinal disturbances (e.g., IBS) by inhibiting Ca^2+^ influx into intestinal smooth muscle cells [40]. Dicetel also inhibits the contractile effect of digestive hormones and proinflammatory mediators such as cholecystokinin, gastrin, and substance P. These metabolites play a key part in the contraction of intestinal smooth muscles, and are linked to defecation-associated abdominal pain and discomfort in patients with IBS.

**Fig. 5:**
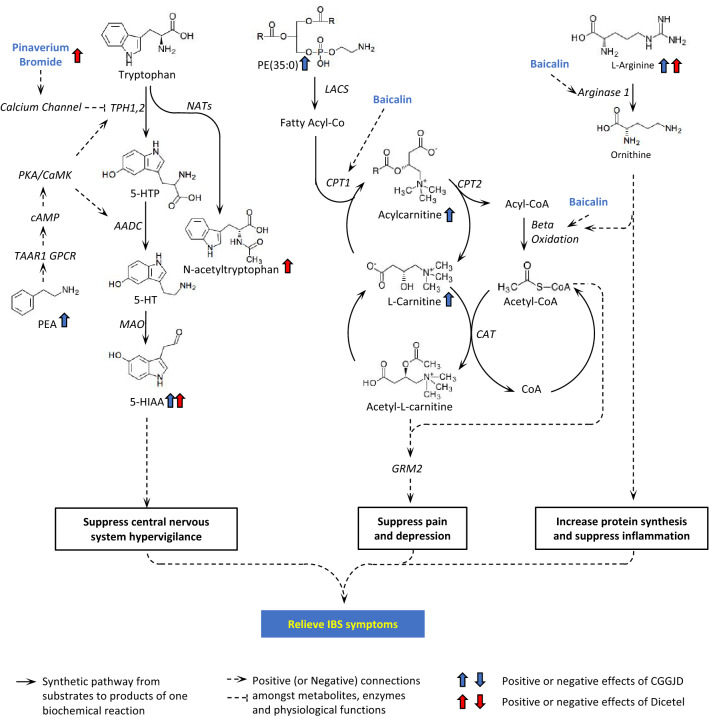
Metabolism and related metabolic processes. Solid arrows denote the positive connections of substrate and products of a single biochemical reaction. Dotted arrows denote positive connections among metabolites, enzymes, and physiological functions. Blue arrows denote the concentration change of metabolites in the CGGD group. Blue arrows denote the concentration change of metabolites in the Dicetel group. The direction of arrows denotes the change in trend

The beneficial effect of Dicetel arose from two biological pathways. First, Dicetel improved tryptophan metabolism, especially the production of 5-HIAA and N-acetyltryptophan (Fig. 5). Tryptophan can be converted to N-acetyltryptophan by N-Acetyltransferase or into 5-hydroxytryptophan (5-HTP) by tryptophan hydroxylase (TPH) 1 and TPH2. Dicetel is a blocker of L-type Ca^2+^ channels, and Ca^2+^ influx through L-type high-voltage-activated calcium channels is essential for full activation of TPH [19, 41]. Hence, inhibiting Ca^2+^ influx using Dicetel would lead to more N-acetyltryptophan. Conversely, 5-HTP can be converted to 5-hydroxytryptamine (5-HT). Once bound to target receptors or taken-up by 5-HT transporters, internalized 5-HT can be metabolized by monoamine oxidase, thereby leading to 5-HIAA generation. Often, the 5-HIAA concentration is used to detect changes in the whole-body 5-HT level [42]. An increased 5-HIAA level cannot be explained directly by the negative influence on TPH by Dicetel. Another compensatory mechanism (e.g., suppressed 5-HT internalization by cells) may also lead to an increased plasma level of 5-HIAA. The Ca^2+^-channel blockers isradipine and darodipine can increase the 5-HIAA: 5-HT ratio in mouse brains [43].

A low 5-HT level can cause depression [44], which is one of the causes leading to IBS. IBS has been postulated to be the most clinically important peripheral disease associated with 5-HT levels [45]. In addition, 5-HIAA and N-acetyltryptophan cause cell toxicity at high concentrations. 5-HIAA can inhibit the growth of the ovary cells of Chinese hamsters [46] and suppresses normal neuronal function by inducing production of oligomerized alpha-synuclein [47]. N-acetyltryptophan is classified as a “uremic toxin” if present in high abundance in sera or plasma [48, 49]. N-acetyltryptophan has been identified as a catabolite of tryptophan generated by the gut microbiota. After absorption through the intestinal epithelium, tryptophan catabolites enter the bloodstream and are excreted subsequently in urine [50]. Uremic toxins are a diverse group of endogenously produced molecules that, if not cleared appropriately or eliminated by the kidneys, can cause kidney damage, cardiovascular disease, and neurological deficits [51]. The overall negative influence of Dicetel on neurons may suppress IBS-related CNS hypervigilance, and improve GI motility and secretion of the enterochromaffin cells [52, 53].

Apart from tryptophan metabolism, Dicetel can alter the arginine level in blood, which can increase protein synthesis and suppress inflammation. These actions also help to relieve IBS symptoms [54].

### Potential pharmacological mechanism of action of CGGD

CGGD has been used in the treatment of liver/spleen deficiency, abdominal pain, and diarrhea [26]. The potential pharmacological mechanism of action of CGGD is more extensive and complex than that of Dicetel. The beneficial effect of CGGD by regulating three biological pathways. Notably, in addition to increased metabolism of tryptophan and arginine (also documented for Dicetel), CGGD improved carnitine-mediated lipid metabolism.

With respect to tryptophan metabolism, CGGD could increase the blood level of PEA, which functions as a neuromodulator or neurotransmitter [55]. PEA is a direct stimulator of 5-HT biosynthesis, thereby regulating GI transit and colonic secretion in vivo and ex vivo. PEA can improve depression [56, 57]. It can activate the G-protein coupled receptor trace amine-associated receptor 1 (TAAR1) which, in turn, mediates 5-HT biosynthesis by stimulating TPH1/ amino acid decarboxylase activities. Zhai and colleagues [58] showed that PEA production stimulates 5-HT biosynthesis to accelerate GI transit via a TAAR1-dependent mechanism. They demonstrated that PEA could relieve constipation [58]. CGGD also increases the blood level of 5-HIAA, which can suppress CNS hypervigilance. baicalin and Chinese thorowax exert positive influence on the concentration of mono amines, including PEA and 5-HT, as reported in the previous study [59], but the exact biological mechanism is under investigation. It is speculated that the Ca^2+^ present in oyster shells can also promote tryptophan to be converted to 5-HTP by TPH1/2 [60, 61], thereby resulting in increased metabolic output [62].

The overall effect of CGGD seemed to increase the availability of acetyl groups, which can suppress pain and depression. L-carnitine is transported across cell membranes primarily by two organic cation transporters (OCTNs): OCTN1 and OCTN2. Once inside a cell, the main physiological function of L-carnitine is to shuttle long-chain fatty acids across mitochondrial membranes with the aid of carnitine palmitoyl transferase 1 (CPT1). The latter is located on the internal side of the external mitochondrial membrane and converts activated fatty acyl-coenzyme A (acyl-CoA) from acyl-CoA to acyl-carnitine. Carnitine translocase exchanges acyl-carnitine for carnitine from the matrix via the internal mitochondrial membrane. On the internal side of the inner mitochondrial membrane, CPT2 catalyzes acyl-CoA synthesis from acylcarnitine and a matrix pool of CoA [71]. Acyl-CoA is processed by beta-oxidation to produce energy in the form of adenosine triphosphate [63]. As demonstrated in vitro and in vivo, S. baicalensis and baicalin can activate CPT directly and accelerate the β-oxidation of lipids [24, 64, 65]. CGGD increases the blood level of long-chain lipids such as PE(35:0) and PC(40:7), as well as acylcarnitine. Acetyl-CoA and acetyl-L-carnitine are important providers of acetyl groups. The latter can activate the glutamate receptor metabotropic 2 gene through epigenetic regulation to suppress pain and depression [66, 67]. Studies [67, 68] have shown that carnitine supplementation can improve the depressive state of male patients suffering from uremia and cancer patients. In addition, the root of Trichosanthes species and oyster shells can supplement levels of lysine and aspartate as synthetic substrates of carnitine, which can increase the carnitine content directly [69, 70]. Therefore, combining our results with previous studies, we speculated that CGGD might also exert its therapeutic effect through the carnitine-mediated lipid metabolism.

CGGD can also increase the blood level of arginine, which can activate adenosine monophosphate kinase, then stimulating fatty-acid oxidation in skeletal muscle and glucose uptake in muscles [56, 57]. This phenomenon can be explained (at least in part) by the existence of baicalin. Baicalin can increase expression of arginase-1 [71], which converts arginine into ornithine. Ornithine has been shown to have a role in regulation of cellular immunity in the micro-environment [72], and its metabolites can stimulate protein synthesis and suppress inflammation. Arginine and its metabolites also have important roles in regulation of esophageal, gastric, and intestinal motility [58].

In fact, phytogenic baicalin, a dominant flavonoid isolated from the roots of Scutellaria baicalensis Georgi, is currently used as a promising complementary agent for prevention and treatment of IBS, as well as other intestinal and neurological diseases based on its neuroprotective, anti-inflammatory, and regulating intestinal flora effects [73–75]. Lei and his colleagues found that baicalin could significantly increase the serum levels of 5-HT in the IBS model mice group compared with the control group [76]. In terms of IBS-related phenotype, after IBS induction modeling, the Bristol fecal character score and abdominal withdrawal reflex (AWR) score were significantly elevated in IBS model group (Bristol fecal character score: 6.40 ± 0.28, AWR score: 2.55 ± 0.21) compared with the health control group (Bristol fecal character score: 3.55 ± 0.45, AWR score: 1.75 ± 0.24) (P < 0.05). Depression, easy stimulation, loss of appetite, varying degrees of loose stools and body hair contaminated by loose stool were observed to occur in the IBS group. After baicalin treatment, symptoms in the IBS group were alleviated. Besides, there was a significant decrease in the Bristol fecal character score and AWR score in the baicalin treatment group (Bristol fecal character score: 5.28 ± 0.39, AWR score: 2.09 ± 0.35, P < 0.05, respectively) compared with the IBS model group compared with IBS model group (Bristol fecal character score: 6.35 ± 0.36, AWR score: 2.67 ± 0.31, P < 0.05, respectively) [76].

In addition to baicalin, other ingredients also had beneficial effects against IBS. First, Rhizoma Zingiberis can supplement ergothioneine directly [77]. According to Cheah and colleagues, ergothioneine can relieve the symptoms of several cognitive diseases [78]. Fond and coworkers have suggested that ergothioneine expression through OCTN1 transporters can protect the nervous system from oxidative stress, maintain energy reserves, provide nutritional and neuroprotective factors, and inhibit abnormal brain excitation, combined with the risk factors of IBS [79], which is also an important reason to relieve patients' symptoms. Second, Radix Glycyrrhizae Preparata also has roles in treating depression [59] and IBS [80].

Collectively, combined with the risk factors of IBS, CGGD can improve the mental state of patients by regulating lipid metabolism. We elucidated the pharmacological mechanism of action of CGGD in regulating lipid metabolism through network pharmacology and lipidomics. This strategy may contribute to the discovery of new drugs and clinical application of CGGD in IBS as well as diseases associated with disorders of lipid metabolism.

### Limitations

Our study has several limitations, which can be explored in future research. First, the sample size was relatively small for such complicated disease and syndrome. A multi-center study on a larger scale is required to provide stronger and more precise metabolomics results.

Secondly, other potential intrinsic and environmental factors that can influence IBS were not assessed in this study. What’s more, only positive results were compared with other positive results from the literature. The metabolites between healthy human and patients with IBD should be further explored, which is necessary for better evaluation of the therapeutic effects of two drugs based on metabolomics. Moreover, the therapeutic effects and biomolecular mechanism of CGGD in treating IBS should been further clarified in the future experiments.

## Conclusions

CGGD was more efficacious than Dicetel for treating IBS according to metabolomics analysis. Dicetel could induce tryptophan metabolism leading to increased blood levels of N-acetyltryptophan and 5-HIAA. Dicetel could also improve arginine metabolism. These Dicetel-related biological pathways could suppress CNS hypervigilance and inflammation and increase protein synthesis. CGGD elicited similar changes to pathways involving acetyltryptophan and 5-HIAA, but also induced carnitine-mediated lipid metabolism to suppress pain and depression. The most efficacious active ingredient of CGGD may be baicalin.

## Supplementary Information

**Additional file 1: Table S1.** Symptom index score. The table contained patient’s symptom index score under CGGD and Dicetel. The figure for TCM criteria comprises: pain or swelling of the chest; abdominal distension or pain; fecal abnormalities; dry throat with a bitter taste; indigestion and anorexia; fatigue; tongue quality. The figure for Rome IV criteria comprises: abdominal pain, abdominal pain days, degree of abdominal pain discomfort, satisfaction of stool condition, inconvenience of intestinal symptoms on life.

**Additional file 2. MS/MS spectral information of significant metabolites.** The table included details for the identification results including MS/MS spectral information (MS1 isotopic spectrum and MS/MS spectrum in column K and L separately).

**Additional file 3. Total ion chromatogram of different stages.** The figure included total ion chromatogram of D0, D30, D37, D67, and QC in this file.

**Additional file 4. Codes for statistical analyses.** The codes in this file uploaded was used for carrying out statistical analyses.

**Additional file 5. Codes for drawing Figure 3.** The codes in this file uploaded was used for drawing our Figure 3.

**Additional file 6. Codes for drawing Figure 4.** The codes in this file we uploaded is used for drawing our Figure 4.

## Data Availability

The data generated in this study are available from the corresponding author upon request.

## References

[1] Ford AC, Lacy BE, Talley NJ (2017). Irritable bowel syndrome. N Engl J Med.

[2] Drossman DA, Hasler WL (2016). Rome IV—functional GI disorders: disorders of gut–brain interaction. Gastroenterology.

[3] Ford AC, Moayyedi P, Lacy BE (2014). American College of Gastroenterology Monograph on the management of irritable bowel syndrome and chronic idiopathic constipation. Am J Gastroenterol.

[4] Lacy BE, Mearin F, Chang L (2016). Bowel disorders. Gastroenterology.

[5] Palsson OS, Whitehead W, Törnblom H (2020). Prevalence of rome IV functional bowel disorders among adults in the United States, Canada, and the United Kingdom. Gastroenterology.

[6] Canavan C, West J, Card T (2014). Review article: the economic impact of the irritable bowel syndrome. Aliment Pharmacol Ther.

[7] Chen G, Xie X, Peng C (2021). Treatment of irritable bowel syndrome by CHINESE medicine: a review. Chin J Integr Med.

[8] Lovell RM, Ford AC (2012). Global prevalence of and risk factors for irritable bowel syndrome: a meta-analysis. Clin Gastroenterol Hepatol.

[9] Zhang L, Duan L, Liu Y (2014). A meta-analysis of the prevalence and risk factors of irritable bowel syndrome in Chinese community. Zhonghua Nei Ke Za Zhi.

[10] Zhu J-J, Liu S, Su X-L (2016). Efficacy of Chinese herbal medicine for diarrhea-predominant irritable bowel syndrome: a meta-analysis of randomized, double-blind, placebo-controlled trials. Evid-Based Complement Altern Med.

[11] Long Y, Huang Z, Deng Y (2017). Prevalence and risk factors for functional bowel disorders in South China: a population based study using the Rome III criteria. Neurogastroenterol Motil.

[12] Holtmann GJ, Ford AC, Talley NJ (2016). Pathophysiology of irritable bowel syndrome. Lancet Gastroenterol Hepatol.

[13] Wall GC, Bryant GA, Bottenberg MM (2014). Irritable bowel syndrome: a concise review of current treatment concepts. World J Gastroenterol.

[14] Radovanovic-Dinic B, Tesic-Rajkovic S, Grgov S (2018). Irritable bowel syndrome—from etiopathogenesis to therapy. Biomed Pap Med Fac Univ Palacky Olomouc Czech Repub.

[15] Adriani A, Ribaldone DG, Astegiano M (2018). Irritable bowel syndrome: the clinical approach. Panminerva Med.

[16] Corsetti M, Whorwell P (2016). Novel pharmacological therapies for irritable bowel syndrome. Expert Rev Gastroenterol Hepatol.

[17] Alammar N, Stein E (2019). Irritable bowel syndrome: what treatments really work. Med Clin North Am.

[18] Surdea-Blaga T, Baban A, Nedelcu L, Dumitrascu DL (2016). Psychological Interventions for Irritable Bowel Syndrome. J Gastrointestin Liver Dis.

[19] Malysz J, Farraway LA, Christen M-O, Huizinga JD (1997). Pinaverium acts as L-type calcium channel blocker on smooth muscle of colon. Can J Physiol Pharmacol.

[20] Baumgartner A, Drack E, Halter F, Scheurer U (1985). Effects of pinaverium bromide and verapamil on the motility of the rat isolated colon. Br J Pharmacol.

[21] Quigley EMM, Fried M, Gwee K-A (2016). World Gastroenterology Organisation Global Guidelines Irritable Bowel Syndrome: a global perspective update September 2015. J Clin Gastroenterol.

[22] Leung WK, Wu JCY, Liang SM (2006). Treatment of diarrhea-predominant irritable bowel syndrome with traditional Chinese herbal medicine: a randomized placebo-controlled trial. Am J Gastroenterol.

[23] Teschke R, Wolff A, Frenzel C (2015). Herbal traditional Chinese medicine and its evidence base in gastrointestinal disorders. World J Gastroenterol.

[24] Bi Z, Zheng Y, Yuan J, Bian Z (2018). The efficacy and potential mechanisms of Chinese herbal medicine on irritable bowel syndrome. CPD.

[25] Xu G-Z, Xue Y, Wei S-Q (2020). Valproate reverses stress-induced somatic hyperalgesia and visceral hypersensitivity by up-regulating spinal 5-HT2C receptor expression in female rats. Neuropharmacology.

[26] Yang X (2009). Syndrome differentiation and treatment of Taiyang disease in Shanghan Lun. J Chin Integr Med.

[27] Hollywood K, Brison DR, Goodacre R (2006). Metabolomics: current technologies and future trends. Proteomics.

[28] Xuan Q, Ouyang Y, Wang Y (2020). Multiplatform metabolomics reveals novel serum metabolite biomarkers in diabetic retinopathy subjects. Adv Sci.

[29] Camilleri M (2020). Irritable bowel syndrome: straightening the road from the Rome criteria. Neurogastroenterol Motil.

[30] Xiaoyu Zheng, et al (2002) Clinical research of new Chinese medicine and the consensus of TCM Diagnosis. China medical scinece press

[31] Spleen and Stomach diseases branch of Chinese Society of Traditional Chinese Medicine. Consensus of experts on diagnosis and treatment of irritable bowel syndrome in Traditional Chinese Medicine. J Tradit Chin Med. 2017;58(18):1614–1620.

[32] Deng Y, Yao H, Chen W (2020). Profiling of polar urine metabolite extracts from Chinese colorectal cancer patients to screen for potential diagnostic and adverse-effect biomarkers. J Cancer.

[33] Gika HG, Theodoridis GA, Wingate JE, Wilson ID (2007). Within-day reproducibility of an HPLC-MS-based method for metabonomic analysis: application to human urine. J Proteome Res.

[34] Tan Y, Yin P, Tang L (2012). Metabolomics study of stepwise hepatocarcinogenesis from the model rats to patients: potential biomarkers effective for small hepatocellular carcinoma diagnosis. Mol Cell Proteomics.

[35] Li M, Chen J, Deng Y, et al (2021) Risk prediction models based on hematological/body parameters for chemotherapy-induced adverse effects in Chinese colorectal cancer patients. Support Care Cancer 29:7931–7947. 10.1007/s00520-021-06337-z. 10.1007/s00520-021-06337-z 34213641

[36] Ritchie ME, Phipson B, Wu D (2015). limma powers differential expression analyses for RNA-sequencing and microarray studies. Nucleic Acids Res.

[37] Li M, Chen J, Liu S (2021). Spermine-related DNA hypermethylation and elevated expression of genes for collagen formation are susceptible factors for chemotherapy-induced hand-foot syndrome in Chinese colorectal cancer patients. Front Pharmacol.

[38] Longstreth GF, Thompson WG, Chey WD (2006). Functional bowel disorders. Gastroenterology.

[39] Camilleri M (2012). Peripheral mechanisms in irritable bowel syndrome. N Engl J Med.

[40] Christen MO (1990). Action of pinaverium bromide, a calcium-antagonist, on gastrointestinal motility disorders. General Pharmacol Vasc Syst.

[41] Maffei ME (2020). 5-Hydroxytryptophan (5-HTP): natural occurrence, analysis, biosynthesis, biotechnology. Physiol Toxicol IJMS.

[42] de Herder WW (2007). Biochemistry of neuroendocrine tumours. Best Pract Res Clin Endocrinol Metab.

[43] Gaggi R, Dall’Olio R, Roncada P, Gianni AM (1995). Effects of isradipine and darodipine on serotonergic system of the rat brain. Pharmacol Biochem Behav.

[44] Firk C, Markus CR (2007). Review: serotonin by stress interaction: a susceptibility factor for the development of depression?. J Psychopharmacol.

[45] Crowell MD, Wessinger SB (2007). 5-HT and the brain-gut axis: opportunities for pharmacologic intervention. Expert Opin Investig Drugs.

[46] Alden N, Raju R, McElearney K (2020). Using metabolomics to identify cell line-independent indicators of growth inhibition for Chinese hamster ovary cell-based bioprocesses. Metabolites.

[47] Jinsmaa Y, Cooney A, Sullivan P (2015). The serotonin aldehyde, 5-HIAL, oligomerizes alpha-synuclein. Neurosci Lett.

[48] Tanaka H, Sirich TL, Plummer NS (2015). An enlarged profile of uremic solutes. PLoS ONE.

[49] Toyohara T, Akiyama Y, Suzuki T (2010). Metabolomic profiling of uremic solutes in CKD patients. Hypertens Res.

[50] Pavlova T, Vidova V, Bienertova-Vasku J (2017). Urinary intermediates of tryptophan as indicators of the gut microbial metabolism. Anal Chim Acta.

[51] Vanholder R, Baurmeister U, Brunet P (2008). A bench to bedside view of uremic toxins. J Am Soc Nephrol.

[52] Ruddell RG, Mann DA, Ramm GA (2008). The function of serotonin within the liver. J Hepatol.

[53] Thijssen AY, Mujagic Z, Jonkers DMAE (2016). Alterations in serotonin metabolism in the irritable bowel syndrome. Aliment Pharmacol Ther.

[54] Witte MB, Barbul A (2003). Arginine physiology and its implication for wound healing. Wound Repair Regen.

[55] Oanca G, Stare J, Vianello R, Mavri J (2017). Multiscale simulation of monoamine oxidase catalyzed decomposition of phenylethylamine analogs. Eur J Pharmacol.

[56] Mulinari S (2012). Monoamine theories of depression: historical impact on biomedical research. J Hist Neurosci.

[57] Violante S, Achetib N, van Roermund CWT (2019). Peroxisomes can oxidize medium- and long-chain fatty acids through a pathway involving ABCD3 and HSD17B4. FASEB J.

[58] Zhai L, Huang C, Ning Z, et al. Phenethylamine-producing gut bacteria induces diarrhea-predominant irritable bowel syndrome by increasing serotonin biosynthesis. Microbiology. 2022. http://biorxiv.org/lookup/doi/10.1101/2022.03.05.483096.

[59] Su GY, Yang JY, Wang F (2014). Antidepressant-like effects of Xiaochaihutang in a rat model of chronic unpredictable mild stress. J Ethnopharmacol.

[60] Kumer SC, Mockus SM, Rucker PJ, Vrana KE (1997). Amino-terminal analysis of tryptophan hydroxylase: protein kinase phosphorylation occurs at serine-58. J Neurochem.

[61] Duchemin AM, Berry MD, Neff NH, Hadjiconstantinou M (2000). Phosphorylation and activation of brain aromatic L-amino acid decarboxylase by cyclic AMP-dependent protein kinase. J Neurochem.

[62] O’Mahony SM, Clarke G, Borre YE (2015). Serotonin, tryptophan metabolism and the brain-gut-microbiome axis. Behav Brain Res.

[63] Fortin G. l-Carnitine and intestinal inflammation. In: Vitamins and hormones. Elsevier, Amsterdam; 2011, pp 353–366 10.1016/B978-0-12-386960-9.00015-0 21419279

[64] Li C, Zhang H, Li X (2020). The mechanism of traditional Chinese medicine for the treatment of obesity. Diabetes Metab Syndr Obes.

[65] Wang Z-Y, Jiang Z-M, Xiao P-T (2020). The mechanisms of baicalin ameliorate obesity and hyperlipidemia through a network pharmacology approach. Eur J Pharmacol.

[66] Adachi T, Fukami K, Yamagishi S-I (2012). Decreased serum carnitine is independently correlated with increased tissue accumulation levels of advanced glycation end products in haemodialysis patients: correlation between carnitine and tissue AGE. Nephrology.

[67] Tashiro K, Kaida Y, Yamagishi S (2017). L-Carnitine supplementation improves self-rating depression scale scores in uremic male patients undergoing hemodialysis. LDDD.

[68] Cruciani RA, Dvorkin E, Homel P (2004). l-Carnitine supplementation for the treatment of fatigue and depressed mood in cancer patients with carnitine deficiency: a preliminary analysis. Ann N Y Acad Sci.

[69] Chen Y, Jiang Y, Liao L (2016). Inhibition of 4NQO-induced oral carcinogenesis by dietary oyster shell calcium. Integr Cancer Ther.

[70] Fang FE, Ng BT, Shaw CP, Wong NSR (2011). Recent progress in medicinal investigations on trichosanthin and other ribosome inactivating proteins from the plant genus Trichosanthes. CMC.

[71] Zhu W, Jin Z, Yu J (2016). Baicalin ameliorates experimental inflammatory bowel disease through polarization of macrophages to an M2 phenotype. Int Immunopharmacol.

[72] Grobben Y, Willemsen-Seegers N, Uitdehaag JCM (2020). High-throughput fluorescence-based activity assay for arginase-1. SLAS Discov.

[73] Liang S, Deng X, Lei L (2019). The comparative study of the therapeutic effects and mechanism of baicalin, baicalein, and their combination on ulcerative colitis rat. Front Pharmacol.

[74] Zhang C-Y-Y, Zeng M-J, Zhou L-P (2018). Baicalin exerts neuroprotective effects via inhibiting activation of GSK3β/NF-κB/NLRP3 signal pathway in a rat model of depression. Int Immunopharmacol.

[75] Ye Y, Huang C, Jiang L (2012). Huanglian-Jie-Du-Tang extract protects against chronic brain injury after focal cerebral ischemia via hypoxia-inducible-factor-1α-regulated vascular endothelial growth factor signaling in mice. Biol Pharm Bull.

[76] Li L, Cui H, Li T (2020). Synergistic effect of berberine-based Chinese medicine assembled nanostructures on diarrhea-predominant irritable bowel syndrome in vivo. Front Pharmacol.

[77] Halliwell B, Cheah IK, Tang RMY (2018). Ergothioneine—a diet-derived antioxidant with therapeutic potential. FEBS Lett.

[78] Cheah IK, Feng L, Tang RMY (2016). Ergothioneine levels in an elderly population decrease with age and incidence of cognitive decline; a risk factor for neurodegeneration?. Biochem Biophys Res Commun.

[79] Fond G, Loundou A, Hamdani N (2014). Anxiety and depression comorbidities in irritable bowel syndrome (IBS): a systematic review and meta-analysis. Eur Arch Psychiatry Clin Neurosci.

[80] Shao Y-Y, Guo Y-T, Gao J (2020). Shaoyao-Gancao Decoction relieves visceral hyperalgesia in TNBS-Induced postinflammatory irritable bowel syndrome via inactivating transient receptor potential vanilloid type 1 and reducing serotonin synthesis. Evid-Based Complement Altern Med.

