# Supplementary figures and images for: Chaihu-Guizhi-Ganjiang Decoction is more efficacious in treating irritable bowel syndrome than Dicetel according to metabolomics analysis
Source: Chin Med. 2022 Dec 14;17:139. doi: 10.1186/s13020-022-00695-4 (PMC9749322; doi:10.1186/s13020-022-00695-4)

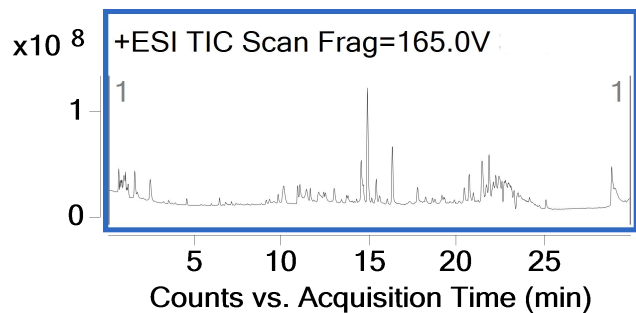

D0

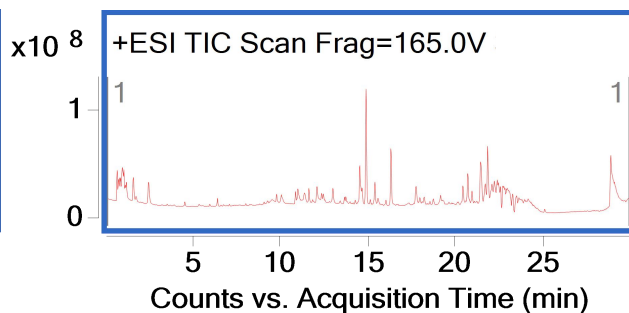

D30

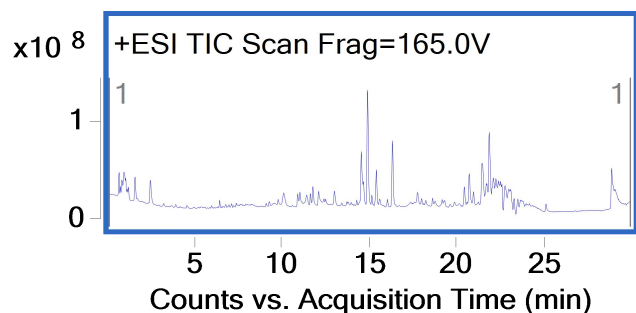

D37

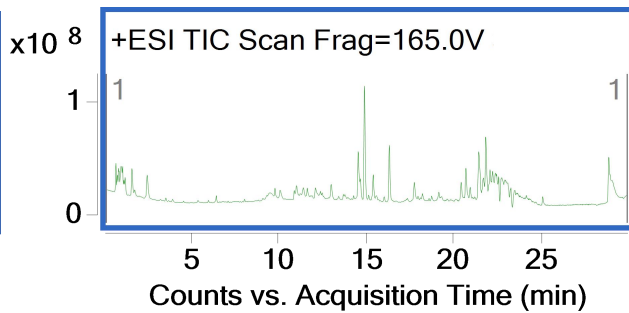

D67

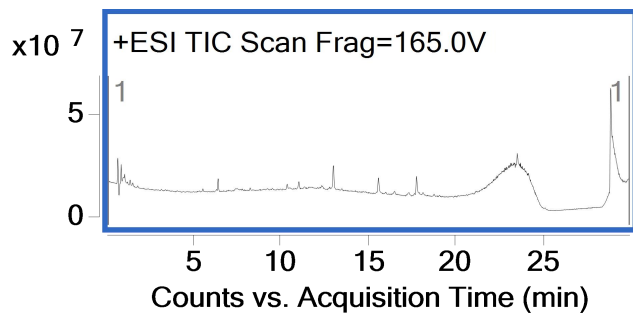

Blank

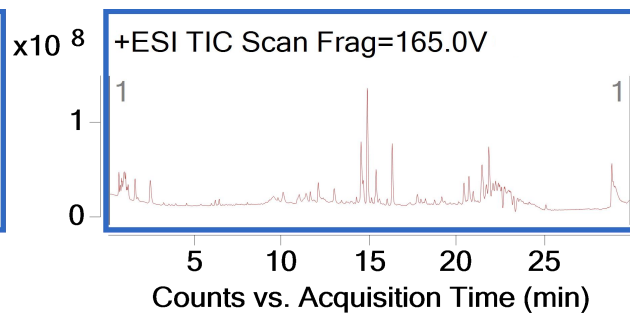

QC

Supplement: Supplementary file 3 — Additional file 3. Total ion chromatogram of different stages. The figure included total ion chromatogram of D0, D30, D37, D67, and QC in this file. [file 13020_2022_695_MOESM3_ESM.pdf]
